# Lymphocyte apoptosis and its association with the inflammatory markers and disease severity in juvenile-onset systemic lupus erythematosus patients

**DOI:** 10.1186/s12969-024-00953-9

**Published:** 2024-01-19

**Authors:** Eman Eissa, Rania Kandil, Dalia Dorgham, Raghda Ghorab, Naglaa Kholoussi

**Affiliations:** 1 Department of Immunogenetics, Human Genetics and Genome Research Institute, National Research Centre, Cairo, Egypt; 2 Rheumatology and Rehabilitation Department, Faculty of Medicine, Cairo University, Cairo, Egypt

**Keywords:** Juvenile-onset systemic lupus erythematosus, Lymphocyte apoptosis, Flow cytometry, Inflammatory cytokines, IL-17, IFN-γ, TNF-α

## Abstract

**Background:**

The defective clearance of apoptotic bodies in juvenile-onset systemic lupus erythematosus (jSLE) potentially leads to the persistence of autoreactive lymphocytes and the perpetuation of the autoimmune response. These factors contribute to the disturbance in lymphocyte apoptosis and show potential as key determinants in the clinical course and severity of jSLE. This study evaluates the role of peripheral blood (PB) lymphocyte apoptosis in prognosis of jSLE and as a predictor for disease activity.

**Methods:**

The study involved 100 jSLE patients and 50 healthy controls. Flow cytometry was used to analyze percentages of lymphocyte apoptosis in PB of all study participants. Plasma levels of pro-inflammatory cytokines were determined using ELISA.

**Results:**

Our results showed that percentages of lymphocyte apoptosis in PB of jSLE patients are significantly higher than those of healthy controls. These percentages are significantly positively associated with disease activity of patients (SLEDAI-2 K). Furthermore, plasma cytokine levels (IL-17, IFN-γ and TNF-α) are significantly elevated in jSLE patients compared to their levels in healthy controls. Also, there are weak significant positive correlations between percentages of PB lymphocyte apoptosis and each of IL-17 and IFN-γ plasma levels in jSLE patients. Moreover, PB lymphocyte apoptosis percentages among jSLE patients are higher in the presence of some clinical and laboratory features than those in their absence.

**Conclusion:**

Peripheral apoptotic lymphocytes could contribute to the prognosis of jSLE and could be used as a predictor for disease activity in jSLE patients.

## Introduction

Systemic lupus erythematosus (SLE) is a systemic autoimmune disease with multiple immune dysregulation mechanisms. Juvenile-onset systemic lupus erythematosus (jSLE) represents a complex and multifactorial autoimmune disorder that predominantly affects children and adolescents, presenting distinct clinical and immunological challenges when compared to its adult-onset counterpart. It differs from adult-onset SLE (aSLE) in its higher disease activity, increased damage at an earlier stage and the need for use of immunosuppressive drugs at more frequent pattern [1]. The etiology of jSLE remains enigmatic, with the interplay of genetic predisposition, hormonal factors, and aberrant immune responses contributing to its pathogenesis. Among the intricate facets of jSLE pathophysiology, dysregulated lymphocyte apoptosis has emerged as a compelling central mechanism in recent years [2].

Lymphocyte apoptosis, a fundamental process of programmed cell death, plays a pivotal role in maintaining immune homeostasis by orchestrating the elimination of autoreactive and senescent lymphocytes, as well as the proper clearance of apoptotic bodies [3, 4]. In jSLE, the delicate balance between apoptosis and cell survival mechanisms, along with the defective clearance of apoptotic bodies, appears to be disrupted. This disruption potentially leads to the persistence of autoreactive lymphocytes and the perpetuation of the autoimmune response. Together, these factors contribute to the disturbance in lymphocyte apoptosis and show potential as key determinants in the clinical course and severity of jSLE [2, 5, 6].

Moreover, mounting evidence suggests that dysregulated lymphocyte apoptosis in jSLE may be closely connected with the pro-inflammatory milieu orchestrated by cytokines [7]. Inflammatory cytokines, including interleukin-17 (IL-17) [8, 9], interferon-gamma (IFN-γ) [10, 11], and tumor necrosis factor-alpha (TNF-α) [12–14], have been implicated as pivotal players in jSLE pathogenesis, contributing to tissue damage and immune dysregulation. The intricate crosstalk between aberrant lymphocyte apoptosis and these inflammatory mediators presents a compelling point for further exploration.

Identification of the molecular processes responsible for leukocyte apoptosis has gained significant consideration [15, 16]. Indeed, numerous research groups have linked various signaling pathway products, such as FasL, NF-κB [17], and Bcl-2 [18], to impaired apoptosis. This potential connection may ultimately contribute to hyperactivity of B and T lymphocytes resulting in excessive accumulation of autoantigens, polyclonal B cell activation and increases in autoantibody production [19, 20].

Lymphocytes themselves have a significant role in controlling the apoptosis of different cell types. For instance, cytotoxic T cells release enzymes like perforin and granzyme B, which can induce apoptosis in target cells. Additionally, T lymphocytes produce Fas ligand (FasL/CD95L), which interacts with Fas (Apo-1/CD95) on epithelial cells, leading to their apoptosis [20].

Unfortunately, the current understanding of immunopathological mechanisms and treatment protocols for jSLE is primarily derived from studies conducted on aSLE patients. This study is dedicated to unraveling the age-specific immunopathogenesis in jSLE and confirming whether it aligns with findings observed in aSLE patients, thereby addressing previous recommendations on this matter [1].

We evaluated the role of peripheral blood (PB) lymphocyte apoptosis in prognosis of jSLE and as a predictor for disease activity. The levels of apoptotic lymphocytes (AL) in PB of jSLE patients was compared to healthy controls. Also, we determined plasma levels of the pro-inflammatory cytokines in jSLE patients in comparison to healthy controls. We correlated PB lymphocyte apoptosis with the clinical manifestations, disease activity and inflammatory markers in jSLE patients.

## Patients and methods

### Ethics approval and consent to participate

This study was approved by the ethics committee of the National Research Centre (NRC), Egypt and has therefore been performed under the ethical standards laid down in the 1964 Declaration of Helsinki and its later amendments. All samples were obtained with the written informed consent of all patients involved in this study before their enrollment.

### Study participants

This study included 50 healthy subjects and 100 juvenile-onset SLE patients (age of onset ≤ 16 years) recruited from Rheumatology and Rehabilitation outpatient clinic, Kasr Al Ainy Hospital, Cairo University from April to December 2022. All patients fulfilled the 2012 Systemic Lupus Collaborating Clinics (SLICC) classification criteria for SLE [21]. Demographic and cumulative clinical manifestations were recorded, and disease activity at the last visit was assessed through the Systemic Lupus Erythematosus Disease Activity Index-2 K (SLEDAI-2 K) [22]. Patients have been divided according to SLEDAI-2 K into active and inactive patients, patients with SLEDAI-2 K ≥ 5 are active while those with SLEDAI-2 K < 5 are inactive.

### Determination of peripheral blood lymphocyte apoptosis using flow cytometry

Apoptosis of lymphocytes was determined in peripheral blood (PB) by Annexin V Apoptosis Detection kit (BD Biosciences, United States) using the BD Accuri™ C6 Flow Cytometer Instrument. The PB was collected from patients and healthy controls in tubes containing EDTA. 50 μL of EDTA‑treated PB were incubated for 30 min at 4 °C in the dark with Annexin V and propodium iodide. The red blood cells were lysed using BD FACS Lysing Solution (Becton Dickinson, USA). The stained cells were then washed and resuspended in buffer solution. Approximately 30,000 stained cells in each sample were analyzed using the BD Accuri™ C6 Flow Cytometer Instrument (BD Biosciences). The lymphocytes were gated by setting the appropriate forward scatter/side scatter axes. Data were acquired, and data analysis was performed by the BD Accuri™ C6 software program.

### Enzyme‑linked immunosorbent assay (ELISA)

Plasma cytokine levels (IFN-γ, IL-17 and TNF-α) of all study subjects were determined using Human ELISA kit (Elabscience, Elabscience Biotechnology Co., Ltd) according to the manufacturer’s protocol.

### Statistical analysis

Data were statistically analyzed using SPSS version 27.0 software (SPSS Inc., Chicago, Illinois, USA). Data were presented as mean ± SD or median (Interquartile range (IQR)). Non-parametric Mann–whitney U test was used to analyze the statistical differences of lymphocyte apoptosis and inflammatory markers between jSLE patients and healthy controls. Association analyses were examined using Spearman correlation analysis. A *P* value of less than 0.05 was considered statistically significant.

## Results

### Baseline manifestations of juvenile-onset SLE patients

The study involved 100 jSLE patients, of whom 96 were females (96%). The mean age at sampling time was 25 ± 8 years, and the median disease duration (IQR) was 140 (112) months. It was observed that 22 (22%) of patients had a family history of SLE and other autoimmune diseases. Cumulative clinical and laboratory patients’ manifestations, disease activity and disease damage and the medications received at sampling time are summarized in Table 1.

**Table 1:** Baseline patients’ Features^a^

	*N* = 100 (%)
***Clinical manifestations***	
Arthritis	72 (72)
Secondary antiphospholipid syndrome	46 (46)
Nephritis	83 (83)
Serositis	29 (29)
Neuropsychiatric	26 (26)
Mucocutaneous	28 (28)
***Immune profile***	
ANA	90 (90)
Anti-ds DNA	81 (81)
aPL	46 (46)
Leucopenia	48 (48)
Hypocomplementemia	50 (50)
***Disease activity and Disease damage at sampling visit***	
SLEDAI-2 K [Median (IQR)]	10 (8)
SDI [Median (IQR)]	2 (2)
***Medications***	
Glucocorticoids	100 (100)
Mycophenolate mofetil	17 (17)
Cyclophosphamide	15 (15)
Hydroxychloroquine	70 (70)
Azathioprine	33 (33)

*Abbreviations*: *IQR* interquartile range, *ANA* anti-nuclear antibodies, *Anti-ds DNA* anti-double stranded deoxyribonucleic acid antibodies, *aPL* antiphospholipid antibodies, *SLEDAI-2 K* Systemic Lupus Erythematosus Disease Activity Index-2 K, *SDI* Systemic Lupus International Collaborating Clinics/American College of Rheumatology Damage Index

^a^Unless indicated, data are presented in number (N) and percentage

### Peripheral blood lymphocyte apoptosis in juvenile-onset SLE patients

**Fig. 1:**
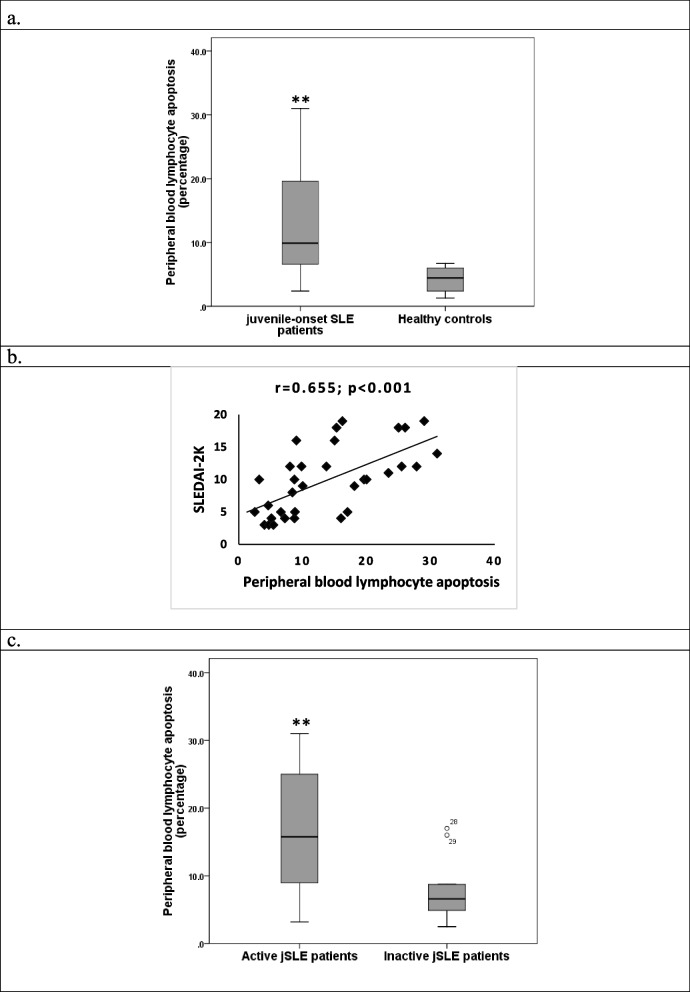
**a**. Comparison of percentages of PB lymphocyte apoptosis between jSLE patients and healthy controls (**: significant at *p* < 0.001, by Mann–Whitney U test). **b** Correlation between percentages of PB lymphocyte apoptosis and SLEDAI-2 K in jSLE patients (*p* < 0.001, by Spearman correlation). **c** Comparison between active and inactive jSLE patients regarding percentages of PB lymphocyte apoptosis (**: significant at *p* = 0.002, by Mann–Whitney U test)

Our findings showed that percentages of lymphocyte apoptosis in peripheral blood (PB) of jSLE patients are significantly higher than those of healthy controls (*p* < 0.001) (Fig. 1a). These percentages are significantly positively correlated with disease activity of patients (SLEDAI-2 K) (*p* < 0.001) (Fig. 1b). Active group of jSLE patients have significantly higher percentages of PB lymphocyte apoptosis than those of the inactive group (*p* = 0.002) (Fig. 1c).

### Plasma levels of inflammatory cytokines in juvenile-onset SLE patients

**Fig. 2:**
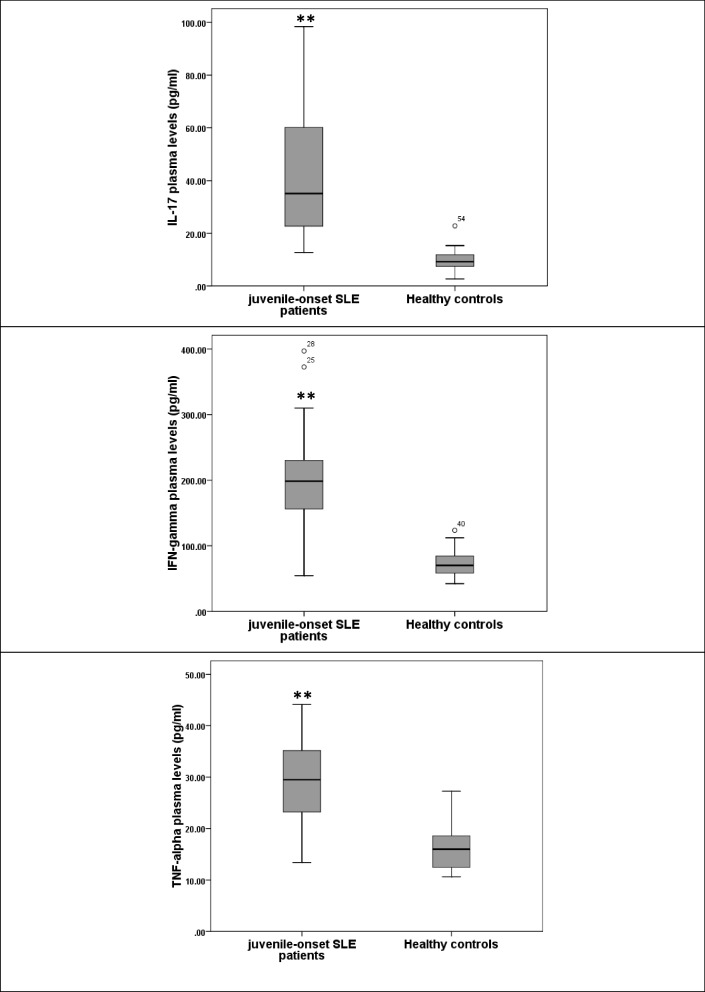
Plasma levels of inflammatory cytokines in juvenile-onset SLE patients compared to healthy controls (**: significant at *p* < 0.001, by Mann–Whitney U test)

We found that plasma levels of inflammatory cytokines (IL-17, IFN-γ and TNF-α) are significantly elevated in jSLE patients compared to their levels in healthy controls (*p* < 0.001) (Fig. 2).

### Correlation between PB lymphocyte apoptosis and inflammatory markers in juvenile-onset SLE patients

Our correlation analysis indicated that there is a weak significant positive correlation between percentages of PB lymphocyte apoptosis and plasma levels of IL-17 (*r* = 0.329; *p* = 0.029) and IFN-γ (*r* = 0.382; *p* = 0.010) in jSLE patients, while no significant correlation was found between levels of TNF-α and percentages of PB lymphocyte apoptosis.

### Comparison of PB lymphocyte apoptosis among jSLE patients based on the presence or absence of clinical and laboratory manifestations

Although there are no statistically significant differences, percentages of PB lymphocyte apoptosis among jSLE patients are higher in the presence of some clinical and laboratory features than those in their absence (Table 2).

**Table 2:** Comparison of PB lymphocyte apoptosis among jSLE patients based on the presence or absence of clinical and laboratory manifestations

Feature		Number of patients *N* = 100 (%)	Percentage of PB lymphocyte apoptosis, median (IQR)	*P* value^*^
Nephritis	present	83 (83)	15 (5.4)	0.981
	absent	17 (17)	10 (11.1)	
Antiphospholipid syndrome	present	46 (46)	9 (6.5)	0.276
	absent	54 (54)	6.2 (3.75)	
Arthritis	present	72 (72)	13.7 (10.9)	0.357
	absent	28 (28)	6.6 (2.7)	
Mucocutaneous	present	28 (28)	15 (18.4)	0.648
	absent	72 (72)	13.5 (5.8)	
Anti-ds DNA	present	81 (81)	13.9 (13)	0.316
	absent	19 (19)	8.4 (2.1)	
Leucopenia	present	48 (48)	19.6 (13.2)	0.683
	absent	52 (52)	8.7 (8.4)	
Hypocomplementenimia	present	50 (50)	10 (9.6)	0.553
	absent	50 (50)	4.6 (8)	

^*^by Mann–Whitney U test

## Discussion

SLE is a systemic autoimmune disease whose pathogenesis implicates many immune mechanisms, interplaying in a rather complex way that has not been fully elucidated yet. The presence of autoantibodies is the hallmark of ‘classical’ SLE that are mainly directed toward nuclear antigens and autoreactive lymphocytes. This applies to most cases of jSLE except those with early onset disease that starts before 5 years of age [2]. The immunopathogenesis is even more complex in jSLE where genetic and hormonal factors contribute more centrally in the pathophysiology [1]. The release of autoantibodies was constantly suggested to be a result from the increase in lymphocyte apoptosis and failure of macrophage clearance of apoptotic cells [6].

This study aimed to evaluate the role of peripheral blood lymphocyte apoptosis in the prognosis of jSLE and as a predictor for disease activity as previously validated and demonstrated for aSLE [6, 23–30]. We also aimed to correlate percentage of AL with pro-inflammatory cytokines and clinical features to eventually reach age-specific laboratory findings elaborating immunopathological mechanisms characteristic for this age group upon which targeted treatment protocols and disease prognosis could address. We found that percentages of AL in jSLE patients were significantly higher than those of healthy controls and they were also significantly positively correlated with disease activity represented as SLEDAI-2 K. In addition, Active group of jSLE patients have significantly higher percentages of PB lymphocyte apoptosis than those of the inactive group. These results resemble findings observed in aSLE in both aspects; increased percentage of AL and their correlation to disease activity [6, 27]. However, other studies that were performed in aSLE showed significance only in the increased AL in aSLE patients while the correlation between their count and disease activity was not significant [28, 30].

The plasma levels of inflammatory cytokines (IL-17, IFN-γ and TNF-α) were significantly higher in jSLE patients in comparison to healthy controls. Similarly, previous observations have shown that IL-17 levels were significantly higher both in jSLE [31–34] and aSLE [35, 36] strongly suggesting its potential as a therapeutic target [37, 38]. Regarding IFN-γ and TNF-α, studies that were performed in pediatric and adolescent patients investigating their role in jSLE have focused on their gene expression levels and polymorphisms rather than their serum or plasma levels; reporting their overexpression in jSLE and correlation with disease activity and clinical manifestations [34]. Polymorphisms in TNF-α was also reported to influence susceptibility to jSLE [39]. Postal et al. found that serum levels of TNF-α was significantly higher in jSLE patients than controls, while serum levels of IFN-γ showed no significant difference. On the other hand, Cavalcanti et al. reported that they did not find significant difference in levels of IL-17, IFN-γ or TNF-α in jSLE [40]. We postulate, considering the smaller sample size included in the latter two studies, that significant differences may have appeared with larger representative samples. This controversy seen in studies done in jSLE patients investigating previous cytokines is not present in those performed in aSLE in which the significant increase in levels of IL-17, IFN-γ or TNF-α has been widely recognized for a considerable time [8, 10–12, 41, 42].

IL-17, IFN-γ and TNF-α are proinflammatory cytokines that are mainly secreted by T helper lymphocytes along with other innate and adaptive immune cells. TNF-α is also primarily secreted by activated macrophages [13], which contribute also to a lesser extent in the production of IFN-γ. IL-17 is specifically produced by Th17 cells derived from naive T cells under the effect of different combinations of other cytokines like TGF-β with IL-21 [43],or IL-6, IL-23, IL-1β and TNF-α [44]. A large fraction of the IL-17 found in SLE patients originates from CD4^−^CD8^−^ double-negative (DN) T cells which also produces IFN-γ. These cells are infrequent in healthy individuals; however, they were found abundantly in PB of SLE patients [45]. IL-17 and IFN-γ impact SLE by modulating autoantibody production through acting directly on B cells [36], or indirectly through recruiting inflammatory cells and stimulating of macrophages, synoviocytes, fibroblasts and neutrophils [37, 46]. Stimulated macrophages and activated T cells produce TNF-α which is known to be a potent apoptotic inducer [13, 14]. The increase in apoptotic cells cause subsequent increase in autoantigen load which indirectly increase autoantibodies [27]. Our findings, which show a positive correlation between plasma levels of IL-17 and IFN-γ and the percentages of AL in peripheral PB of jSLE patients, align with the previous explanations of immunopathogenesis. This provides affirmation for investigating these factors in the specific context of jSLE. Although no significant association was found between the levels of TNF-α and the percentages of AL, its concurrent significant elevation in jSLE patients, along with IFN-γ, rules out the presence of an intrinsic defect in macrophage function as a possible cause for the defective clearance of apoptotic bodies. In accordance with this finding, a prior study has shown that lower ability of macrophage to phagocytose apoptotic bodies was secondary to serum factors rather than a primary defect in macrophages [27]. Complement deficiency was suggested to be among these factors [47]. Notably, hypocomplementemia was observed in fifty percent of our patients.

The main limitations of this study involve the limited number of individuals in each group. Furthermore, the drugs used by patients might have affected the percentages of lymphocyte apoptosis. However, studying the correlation between percentages of lymphocyte apoptosis and disease activity is highly important in exploring their role in the prognosis of lupus.

## Conclusion

To the best of our knowledge, this is the first in vivo case control study that investigated the role of AL in jSLE in practical means that provide age-specific relevance for its prognostic performance in terms of evaluating its significance in differentiating active from inactive disease and furthermore, correlating with other inflammatory cytokines which showed evidence that can prove theory implicating defective macrophage clearance in immunopathogenesis. Further studies are needed to elucidate the sequence of events revealed in the results regarding the interplay between AL and inflammatory cytokines. These studies should determine whether AL is a consequence of systemic inflammation or if it acts as the driving force behind such events, possibly influenced by other serum factors. Identifying the trigger for these events should be the focus of future research aimed at exploring potential therapeutic solutions.

## Data Availability

The datasets used and/or analyzed during the current study are available from the corresponding author on reasonable request.

## Abbreviations

SLE: Systemic lupus erythematosus
jSLE: Juvenile-onset systemic lupus erythematosus
aSLE: Adult-onset SLE
PB: Peripheral blood
SLEDAI: Systemic lupus erythematosus disease activity index
IL-17: Interleukin-17
IFN-γ: Interferon-gamma
TNF-α: Tumor necrosis factor-alpha
FasL: Fas ligand
AL: Apoptotic lymphocytes
SD: Standard deviation
IQR: Interquartile range
ANA: Anti-nuclear antibodies
Anti-ds DNA: Anti-double stranded deoxyribonucleic acid antibodies
aPL: Antiphospholipid antibodies
SDI: Systemic Lupus International Collaborating Clinics/American College of Rheumatology Damage Index
